# Deficits in emotional cognition among individuals with conduct disorder: theoretical perspectives

**DOI:** 10.3389/fpsyt.2024.1507695

**Published:** 2024-12-10

**Authors:** Xue Li, Hui Kou, Taiyong Bi, Zuoyang Peng

**Affiliations:** ^1^ Department of Teaching and Research on Party Building, Guizhou Institute of Public Administration, Guiyang, China; ^2^ Research center of Humanities and Medicine, Zunyi Medical University, Zunyi, China; ^3^ Department of Education, Guizhou Provincial Government, Guiyang, China

**Keywords:** conduct disorder, emotional processing, theoretical perspectives, cognitive deficit, emotional cognition

## Abstract

Conduct disorder refers to a persistent and repetitive pattern of problematic behavior, and is usually accompanied by deficits in cognitive processing of emotional stimuli. Behavioral and physiological correlates of these deficits have been the subject of sufficiently many investigations. Meanwhile, several theories concerning conduct disorder have been developed. However, the validity of these theories to explain the emotional processing deficits among conduct-disordered individuals has not been tested. Here we summarized four relevant theories, including “social-cognitive theory and social-information-processing theory”, “violence inhibition model”, “optimal stimulation/arousal theory and stimulation/sensation seeking theory”, and “fearlessness theory”, and discussed their validity in predicting the emotional processing deficits among conduct disordered individuals. Future directions on the theories and evidence were proposed.

## 1 Introduction

### 1.1 Conduct disorder

Conduct disorder (CD) is defined as a persistent and repetitive pattern of problematic behavior that violates others’ rights or that violates age-appropriate social norms or rules, and is characterized by a pattern of severe antisocial and aggressive behavior (1). CD usually manifests in childhood or adolescence, and is usually associated with delinquent or criminal behaviors (2–4).

It is worth noting that there are two types of CD, the child/early-onset CD (CO-CD) and the adolescent-onset CD (AO-CD), and the mechanisms between them may be different. CO-CD is thought to arise from neurodevelopmental difficulties which play a causal role in the aetiology of their antisocial behavior. CO-CD usually shows verbal intelligence quotient (IQ) deficits. In contrast, AO-CD is thought to arise from the social imitation of deviant peers. Previous studies demonstrated that the gray matter volume (GMV) of the right temporal lobe was reduced among CO-CDs (5), while the cortical thickness in the paralimbic system was found attenuated among adolescents with AO-CD (6). Another study showed that the GMVs of the bilateral amygdala in both CO-CD and AO-CD were reduced while the GMV of the right insular was only reduced in the AO-CD group (7).

## 2 Emotional processing among CD individuals

It is important to study the mechanism behind CD, as a thorough understanding of these processes can contribute to the development of more effective treatments and interventions. This, in turn, can greatly reduce antisocial behaviors for individuals with CD. Furthermore, a deeper understanding of these mechanisms can also aid in the early detection and prevention of CD, thereby reducing the overall societal impact and burden associated with CD. Emotional processing has been considered a core function in social functioning. The deficits in emotional cognition may lead to antisocial behavior and thus be the underlying mechanism of CD.

### 2.1 Deficits in emotional recognition

Emotional recognition is shown impaired among individuals with CD (8). For all basic emotions of happiness, sadness, anger, fear, disgust, and surprise, and for both static and dynamic expressions, children and adolescents with CD were found impaired in emotional recognition (9–11). However, different studies may draw different conclusions on the deficits in specific emotions. For example, some researchers found that individuals with CD showed impairments in the recognition of negative emotions (12), such as anger, disgust, fear, and sadness, but not for happiness and surprise (13). Other researchers found that adolescents with CD had deficits in recognizing anger, surprise, fear, happiness, and sadness but not disgust (14). Among the emotions, the recognition of sadness and fear may be most usually and consistently found impaired among CDs. For example, the recognition of sad and fearful faces was found impaired among children with psychopathic tendencies involved in affective-interpersonal disturbance and impulsive behavior/conduct problems (15). Another study showed that conduct problems were inversely correlated with the recognition of sad and fearful expressions but not happy, angry, disgusted, or surprised expressions among children aged 11-14 years (16). However, with a classification task, it was shown that children with CD tended to incorrectly identify happiness and fear as angry (17), and adolescents with CD were more likely to classify neutral faces as fearful (11). The different findings among studies may result from the different tasks that were adopted. Some researchers adopted the Emotion Hexagon task to assess facial emotion recognition in which participants were required to view six morphed facial expressions continua (9, 14, 16, 18, 19). Some researchers adopted an alternative-forced-choice task in which participants were asked to discriminate facial emotions (10, 11, 13, 20). Some researchers adopted the Diagnostic Analysis of Nonverbal Accuracy to assess the recognition of sadness, happiness, anger, and fear (15). Further studies are required to elucidate the difference between these tasks.

The type of CD is also an influential factor that largely affects the deficits of emotional processing among CD. As we described above, CO-CD arises from a neurodevelopmental deviation, while AO-CD is developed by social imitation from deviant peers. Therefore, individuals with CO-CD may show more severe deficits in emotional recognition. Research revealed that male adolescents with CO-CD showed impairments in recognizing angry, disgusted, and happy facial expressions, whereas those with AO-CD only showed deficits in recognizing fear (21). Besides the developing type, another way to differentiate the type of CD is based on the trait of callous-unemotional (CU). It was proposed that the impairment in emotion recognition among CD was related to CU traits (9). Research revealed that CD with high CU showed deficits in recognizing sad faces (18, 22), while CD with low CU showed deficits in recognizing fearful faces (23). Consistently, evidence illustrated that higher levels of CU traits were associated with better performance in recognizing fearful expressions (10, 22, 23). Neuroimaging evidence indicated that, compared with typically developing controls and CDs with low CU, CDs with high CU showed hyperactivity within the dorsolateral, dorsomedial, and ventromedial prefrontal regions during emotional recognition of angry and fearful faces (24). However, there was also evidence showing little influence of the CU trait on the recognition of emotions (25).

### 2.2 The neural substrates of emotional processing deficits

Cappadocia et al. (26) summarized that individuals with CD exhibit abnormal structures and functions, including reduced P300 amplitude, deactivation in the anterior cingulate cortex, attenuated activation in the left amygdala in response to negative stimuli, reduced volume of the right temporal lobe, lowered serotonin levels, and attenuated autonomic nervous system reactivity. Regarding research concerning facial emotions, CD individuals showed reduced activities in brain regions related to antisocial behavior and amygdala for angry faces when they passively viewed angry, sad, and neutral faces (27). Another study also found reduced activation in the amygdala in response to angry and sad (vs. neutral) faces among CD (28). Consistent with the behavioral evidence, the neural activity deficits of emotional processing are also modulated by the type of CD. First, the CO-CD and AO-CD subgroups showed different neural activities. It was found that, compared with controls and AO-CD, the CO-CD group exhibited reduced activation in the amygdala for sad (vs. neutral) faces (27). Second, CU may also modulate the neural activities for emotional processing. It was found that CD with high CU and low CU showed different response patterns in the amygdala for various emotional faces (29, 30). For example, amygdala responses to fear or distress stimuli decreased among CD with high CU, while they increased among CD with low CU (31). For emotional faces that were presented subliminally, CD with low CU also exhibited greater responses in the right amygdala to fearful faces than CD with high CU (30). Contrary to the reduced activities in the amygdala, CD with high CU showed hyperactivity in the dorsolateral, dorsomedial, and ventromedial prefrontal regions and increased activation in the posterior cingulate and precuneal cortices, compared with CD with low CU, when viewing angry and fearful faces (24). Taken together, these results demonstrated impaired functioning of the amygdala to negative stimuli among individuals with CD, especially those with CO-CD and high CU traits.

In summary, understanding the emotional deficits in individuals with CD holds significant implications. First, it may help develop targeted interventions to improve emotional processing in these individuals. By addressing these deficits early, it may reduce the risk of further antisocial behaviors and promote more adaptive social functioning. Furthermore, it provides insights into educational strategies that could help in the identification and support of high-risk youth, thereby preventing the emergence of more severe behavioral problems. By enhancing the emotional awareness of educators and guardians, these strategies can in turn lead to more effective interventions and support systems.

## 3 Theories concerning the emotional processing deficits

Numerous theories have been proposed to explain behaviors characterized by antisocial tendencies, including aggression. From a biological standpoint, there are the prefrontal cortex dysfunction hypothesis (32) and the optimal arousal theory (33, 34). When considering social factors, there are theories such as the general strain theory (35) and social learning theory (36). Personal factors encompass a range of theories, including cognition-related theories, such as the social information processing theory (37), social cognitive theory (38), and violence inhibition model (39, 40); affect-related theories, like the emotion dysregulation theory (41) and integrated emotion systems model (42); and personality-related theories, including the fearlessness theory (43), stimulation-seeking theory (44, 45) and others. Additionally, there are comprehensive theoretical frameworks, such as the general aggression model (46, 47) and the biopsychosocial model (48).

However, as we discussed above, emotional processing deficits may be a core factor contributing to the antisocial behaviors in individuals with CD and acquired attention from more and more researchers. Among the aforementioned theories, the social cognitive/social information processing theory, the violence inhibitory model, the optimal stimulation/arousal theory, the stimulation/sensation seeking theory, and the fearlessness theory are particularly well-suited for elucidating the underlying mechanisms. Therefore, we aim to discuss the connection of these four theories with the emotional processing of CD individuals in detail.

### 3.1 Social-cognitive theory and social-information-processing theory

The social-cognitive theory primarily includes two basic hypotheses: belief and belief-driven social information processing (38). Belief refers to an idea or proposition that is unrelated to actual objective truth but is accepted for being considered to be true (49). Everyone has developed interconnected belief networks, and this highly related cluster of beliefs is often called schema (50, 51). Schema is not developed based on truth and objectivity but is a subjective cognitive structure that is greatly influenced by one’s past knowledge and experience. For example, self-schema is cognitive generalizations about the self that are derived from early experience. Information in a schema can be used to perceive, describe, interpret, and predict complex society. The schemata about oneself and society will, to a large extent, automatically guide a person to understand the behavior of himself/herself and others (51). Schneider (52) proposed that social information processing is fundamentally top-down or schema-driven. To improve the processing efficiency of social information, people generally do not have sufficient time and resources to understand or interpret their own and others’ behaviors in a logical, thoughtful, and careful way. Instead, they rely on their schemata to automatically and unconsciously understand the ambiguous social environment.

According to the social-information-processing theory (37), an individual’s behavioral response to social stimuli could be viewed as the result of a series of steps for social information processing. The first step is encoding related aspects of social stimuli from sensory input, for example, selective attention to social cues (e.g., facial expressions) and the storage of cue information in short-term memory. The second step is mental representation, in which individuals keep a subjective interpretation of a stimulus, rather than the stimulus itself, in their memory. The third step is response accessing which could be one or more behavioral and emotional responses triggered by mental representation. For example, if one interprets the other’s angry expression as hostility or a threat to himself, he/she may respond in a hostile, aggressive way. However, not all accessing responses will be executed, and thus the fourth step is response evaluation and selection, which reflects the decision-making process. Reactions can be evaluated in terms of moral acceptability and expected outcomes. The final step is the enactment, which translates the selected response into actual action. This theory primarily focuses on the mental process which leads to maladapted behavioral responses to social stimuli (37). Sestir and Bartholow (53) proposed that the way humans perceive, process, store, and retrieve information shapes their aggression. In a word, social information processing links schemata to behaviors, while schemata link early experiences to future social information processing in certain situations. One’s adverse experience shaped the maladaptive schemata which in turn may have an adverse impact on his/her social information processing, and then this maladaptive processing may lead to problematic behaviors. A previous study revealed that the negative social cognitive bias played a mediating role in the relationship between exposure to community violence and aggression, which supported this theory (54).

In conclusion, according to the social-cognitive theory and the social-information-processing theory, the schema is the knowledge structure shaped by one’s unique experience and can automatically guide his/her social information processing which involves encoding, mental representation, response accessing, response selection, and enactment (37, 38, 55, 56). Previous studies revealed that aggressive or violent individuals were more likely to hold or endorse aggression-related schema (57–59), and that these individuals usually showed a pattern of biased processing, for example, the negative social-cognitive bias (54), the attention bias towards hostile social cues (60–62), selective recall for hostile social cues (63) and the hostile attribution bias (63, 64). The social-cognitive theory and the social-information-processing theory can be extended to describe and interpret CD. The logic is that, if a specific information-processing action is associated with a deviant behavior (e.g., assault or aggression), a general processing pattern (e.g., attentional bias to hostile cues) will be relevant to a general behavioral pattern (e.g., CD) (37). Because of exposure to childhood abuse or maltreatment (65–69) and exposure to aggressive/deviant models or peers (70–72), individuals with CD develop a maladapted or distorted schema, which leads them to preferentially encode hostile or threatening cues, to mentally interpret or represent social cues as threats, to easily access aggressive responses and finally to engage in aggressive behavior.

The two theories explain how maladaptive cognitive processing such as emotional processing impact behavioral outcomes. CD individuals exhibit difficulties in recognizing and interpreting emotional expressions, which can lead to misinterpretations of others’ intentions and subsequent aggressive reactions. These deficits in emotional recognition are believed to be a significant contributor to the development and maintenance of CD symptoms. However, more caution is needed when using social information processing theory to explain emotional processing deficits in CD individuals. Although social information processing deficits like hostile attribution bias are often thought to play a significant role in fostering antisocial and aggressive behaviors, it remains somewhat unclear how broader impairments in the processing of emotional faces might contribute to such problematic behaviors. For example, a recent study has indicated that male adolescent delinquents with CD exhibit deficits in attentional orientation to hostile (angry) and threatening (fearful) faces, rather than sad and happy faces (73), which seems to be inconsistent with the social cognitive theory and social information process theory. To better understand how deficits in emotional processing contribute to problematic behaviors, a negative interpretation bias toward facial emotions could be crucial. Individuals with CD were more likely to identify happiness and fear as angry (17), and to classify neutral faces as fearful (11). Some studies have revealed that emotional recognition training can significantly improve negative interpretation of emotional faces (e.g., recognize angry faces as happy ones), which in turn reduces hostility, state anger, aggression, and behavioral problems (74–76). Additionally, the impact of impairment in emotional processing on behaviors among CD individuals might be modulated by some personal traits such as CU traits (9, 10, 18, 22, 23). It is possible that although the cues of fear or sadness expressed by a victim might stop the misbehavior of individuals without CU traits, the same cues might has no effect on individuals with CU traits. Therefore, understanding the nuances of these emotional processing deficits is crucial for developing effective interventions to reduce antisocial and aggressive behaviors.

### 3.2 Violence inhibition model

The violence inhibition model comprises at least two stages, namely emotional perception (including empathy) and movement cessation, and typically the organism learns to regulate aggression by perceiving others’ distress (39, 40). When the distress during non-verbal communication (e.g., sad expression) is activated, humans have a cognitive mechanism that can initiate their withdrawal responses. This is a schema that can make individuals tend to withdraw from aggression and can mediate the relationship between the distressed cues and aggression. A previous study revealed that distressed cues (e.g., a sad face) can indeed terminate children’s aggression (77). According to this model, it can be assumed that individuals with CD have defects in emotional processing for distressing stimuli.

At the first stage of this model (emotional perception), the distressing stimuli lose their value because individuals with CD have difficulty in normally encoding affective stimuli. Evidence showed that adolescents with CD usually exhibited lower empathy and corresponding abnormal neural activities in emotion-related areas such as the amygdala and insula, indicating difficulty in emotionally arousing among them (8, 78–80). Direct measurements of the emotional responses and physical responses to affective stimuli also revealed the defect. For example, boys with CD reported lower levels of emotional responses to unpleasant stimuli (81), and showed reduced electrodermal responses to the distress cues (e.g. a crying face) compared with threatening cues (e.g. an angry face) (82). Consistent with these findings, it was shown that boys with conduct problems needed more stages before they successfully recognized sad expressions, and that they were more likely to recognize the fearful expression as another emotion, which indicated encoding defects for distressing emotions (83).

At the second stage (action stop), the model proposes that individuals with CD have defects in executive function related to violence inhibition (e.g. error monitoring and response inhibition). Previous studies revealed that CD was correlated with lower executive cognitive function capacity in female adolescents (84), as well as deficits in response inhibition (85), and individuals with CD had deficits in inhibitory control (13, 86). Evidence showed that the cingulate, insular, and prefrontal cortices were associated with and modulate aggression (87). Within this framework, the dorsolateral prefrontal cortex (dlPFC), as the rostral component of the fronto-parietal executive function network, plays a crucial role in regulating impulsivity and inhibiting aggression. Previous studies demonstrated that CD symptoms were associated with a reduced structure in the prefrontal and anterior cingulate cortex (88). Furthermore, individuals with CD demonstrate significant differences in effective connectivity within the inhibition control network when compared to healthy controls (89). Taken together, the violence inhibition model predicts that both deficits in emotional perception and impulsive inhibition result in conduct problems.

Similarly, the I^3^ model also emphasizes that the three distinct processes of instigation, which encompass immediate environmental stimuli (e.g., provocation), along with impellance and inhibition, encompassing situational or dispositional qualities, interact and further produce aggression (90, 91). According to this theory, aggressive behavior is not simply a response to provocation (instigation) but is also influenced by the individual’s ability to inhibit such impulses (inhibition). By integrating this perspective, the violence inhibition model can be refined to account for the dynamic nature of aggressive responses.

In summary, the violence inhibition model divides the influence of emotional processing on behavioral outcomes into two distinct and separate stages, which provides a clear explanation of how emotional cognitive processing plays a role in behavioral outcomes. By breaking down the process into these two stages, the model offers a nuanced understanding of the relationship between emotional cognition and the subsequent behavioral responses that are shaped by it. However, the model has limitations when explaining the deficits in emotional processing, because the impairments may be confined specifically to emotional cognition in response to distressing stimuli, rather than being a general issue across all emotional processing domains.

### 3.3 Optimal stimulation/arousal theory and stimulation/sensation seeking theory

Quay (92) proposed that both the prolonged absence of stimulation and the prolonged presentation of persistent monotonic stimulation are unpleasant and can trigger greater motivation. According to the optimal stimulation/arousal theory, everyone has an optimal arousal level of stimulation, and a large deviation from this optimal level will lead to an aversive state, and then make the individual exhibit compensatory behaviors to recover the optimal arousal level by enhancing or reducing current arousal level (33, 34). Thus, sensory overload generally leads to physical or mental withdrawal and limits sensory input, while sensory deprivation generally leads to increased physical or mental activity.

Regarding individuals with CD, they are usually under-aroused, and thus exhibit the state of stimulation/sensation seeking. Previous studies revealed that children with CD showed reduced baseline or resting heart rates, skin conductance, and electrodermal activity, as well as lower task electrodermal activity (26, 93). Besides, studies also showed that male adolescents with aggressive CD showed attenuated respiratory sinus arrhythmia (RSA), fewer skin conductance responses, and a longer pre-ejection period (PEP) that was an index of sympathetic (beta-adrenergic) activity at baseline (94), and that female adolescents with CD had lower morning plasma cortisol levels (95). Moreover, children with CD showed lower autonomic nervous system (ANS) and hypothalamic-pituitary-adrenal (HPA) axis activity (93, 95–97). These physiological indicators were not only reduced among CD individuals, but also associated with CD symptoms. For example, cortisol stress reactivity and cortisol recovery could negatively predict aggression (98), and declined cortisol stress reactivity could predict CD symptoms (99). Taken together, it suggests that individuals with CD are under-aroused. However, it should also be noted that some evidence may not support this proposition (100).

According to the optimal stimulation/arousal theory and the stimulation/sensation seeking theory, it can be assumed that individuals with CD have a low level of physiological arousal less than the optimal arousal level, so they are more likely to exhibit hyperactivity, such as social rules violation or illegal behaviors, to increase their physiological arousal levels and make up for the aversive state (33, 34, 45, 92, 101). Individuals with CD have greater needs for external/behavioral sensory-seeking than internal/mental sensory-seeking, and they may be raised in environments that are difficult to meet their sensory needs in a socially acceptable way, which forces them to meet their needs in a way that violates social or legal norms. Some researchers found that higher heart rate reactivity was associated with conduct problems, for example, higher heart rate levels in response to frustration and provocation (97, 102). Other evidence showed that heart rates were correlated with aggression and rule-breaking, and sensation-seeking played a mediating role in the relationship between heart rates and rule-breaking in male adolescents (103, 104). These results support that antisocial and aggressive behaviors are stimulating for some individuals and are a way to satisfy their stimulation-seeking, implying that violence and aggression are byproducts of stimulation-seeking behaviors.

In the frame of the optimal stimulation/arousal theory and the stimulation/sensation seeking theory, individuals with CD are hard to be aroused and stimulated by emotional stimuli. A lot of studies demonstrated that when viewing emotional stimuli, individuals with CD reported attenuated skin conductance responses (81), reduced temperament skin conductance magnitudes (105), lower startle-elicited blinks (18, 106–108), and lower affect-related activities in the amygdala/hippocampal formation, parahippocampal gyrus, ventral striatum, and anterior and posterior cingulate gyri (109).

In contrast to the violent inhibition model, both the optimal stimulation/arousal theory and the stimulation/sensation-seeking theory indicate that impaired emotional cognition might not be confined solely to the affective valence. It is possible that emotional processing might be affected by the intensity of stimulation, implying that the level of arousal could play a significant role in how emotions are processed. Therefore, while these two theories provide some insights into the nature of emotional processing deficits, they offer limited explanations for emotional processing deficits among individuals with CD. This limitation arises because these theories do not adequately address why individuals with CD process different types of emotions in varying ways.

### 3.4 Fearlessness theory

According to the fearlessness theory, individuals need fear, to a certain extent, to inhibit antisocial and violent behaviors, and the lack of fear for social punishment in childhood may result in poor fear conditioning and inadequate development of conscience (43). Fearlessness in individuals with CD is found to be associated with lower sensitivity to punishment cues (110). As the sensitivity to punishment reflects the behavioral inhibition in the context of aversive outcomes (111), fearless individuals are insensitive to the negative outcomes (e.g. punishment) caused by their aggressive behaviors, which may lead to difficulty in learning to inhibit antisocial and aggressive behaviors. A recent study revealed that youths with CD showed deficits in learning to stimuli evoking punishments (9). Electrophysiological evidence suggested that male adolescents with CD exhibited decreased responses in a gambling task to the loss which implied punishment (112). Therefore, fearlessness may play a crucial role in conduct problems. Previous studies demonstrated that fearless boys were at a high risk of CD (113), and children with increasing conduct problems were characterized by increases in their levels of fearlessness (114). Longitudinal studies revealed that the level of fearlessness for fearful voices at age 2 could not only predict the initial level of conduct problems at age 2 but also predict the persistence of conduct problems between 2 and 8 years of age (115). Another study showed that the fearless temperament at age 2 could even predict conduct problems at age 13 (116).

Moreover, the fearlessness theory also suggests that a low level of arousal under moderate stress may reflect a low level of fear (43, 117), and thus could also indicate weak emotional processing among CD individuals, especially for anger and fear. For example, boys with CD showed reduced corrugator muscle activity, indicating a deficit in perceiving anger when exposed to dynamic angry expressions (118). In addition, they also showed decreased heart rate in response to fear and sadness, which was correlated with CD symptoms (119). Another study revealed that boys with conduct problems were more likely to identify fearful faces as another expression even when the fearful expressions were at full intensity (83). These results indicate that individuals with CD have problems processing threatening stimuli, which may reduce the arousal of fear induced by these stimuli. However, there are few direct examinations of the relationship between fearlessness and cognitive processing of emotional stimuli. One study revealed that fearlessness in individuals with CD was associated with lower sensitivity to aversive stimuli (punishment cues) (110). Future studies are required to further investigate this relationship.

The fearlessness theory posits that individuals with CD exhibit reduced fear responses to aversive stimuli, which may contribute to their increased likelihood of engaging in antisocial behaviors. However, although this theory provides a compelling explanation for certain behaviors in specific contexts, it does not fully account for the complexity of emotional processing deficits observed in these individuals. Similar to the violence inhibition model, the fearlessness theory encounters certain limitations when it comes to comprehensively explaining the emotional processing deficits in these individuals. The impairments might be uniquely restricted to emotional cognition in response to threatening or aversive stimuli. This suggests that although fearlessness may play a role, it is not the only factor influencing the emotional recognition impairments observed in CD. Therefore, although fearlessness may be a significant factor, it is essential to consider a broader range of influences to gain a comprehensive understanding of the emotional processing deficits in CD.

## 4 Prospects

Individuals with CD show significant deficits in processing emotional information. We summarized several theoretical perspectives which could predict and explain the emotional processing deficits, including “social-cognitive theory and social-information-processing theory”, “violence inhibition model”, “optimal stimulation/arousal theory and stimulation/sensation seeking theory”, and “fearlessness theory”. Each theory focuses on a distinct facet that sheds light on why conduct disorder (CD) and aggressive behaviors emerge and develop. From these perspectives, emotional processing is also influenced and impaired among CD individuals. Although these theories may successfully predict the deficits in emotional cognition observed in individuals with CD, the underlying logic and specific predictions can vary considerably from one theory to another. For example, according to the optimal stimulation/arousal theory and stimulation/sensation seeking theory, it is hypothesized that the impaired emotional cognition might not depend on the affective valence, but is a more generalized impairment. In contrast, the fearlessness theory posits that individuals with CD may exhibit specific deficits in cognitive processing related to negative emotions, particularly those that are threatening. Regarding the violence inhibition model, it suggests that the impairments may be restricted to emotional cognition for distressing stimuli, implying a more selective deficit. At last, the social-cognitive theory and the social-information-processing theory predict that individuals with CD will show cognitive biases toward schema-consistent emotion stimuli (e.g. angry expression for aggressive schema). For example, they might exhibit a trend to interpret ambiguous expressions as angry, which aligns with their aggressive schema. This indicates that their emotional processing is biased towards recognizing and responding to emotional cues that reinforce their pre-existing aggressive frameworks. In summary, while each theory offers valuable insights into the emotional processing deficits associated with CD and aggression, they differ in their explanations and predictions. These differences highlight the complexity of emotional cognition in CD and underscore the need for a multifaceted approach when studying and addressing these deficits. Further evidence is required to reconcile different theories and to develop a more unified theory of CD.

Additionally, the type of CD is a crucial factor that modulates the emotional processing among CD, as we discussed above. Further theories may pay more attention to explaining emotional recognition deficits among subtypes of CD. For example, evidence showed that CO-CD was impaired in recognition of angry, disgusted, and happy facial expressions whereas AO-CD was impaired in recognition of fear (21). Neuroimaging evidence suggested that CO-CD exhibited hypoactivity in the amygdala for sad faces, compared with AO-CD (27). The second way to subdivide CD is based on the CU trait. Some researchers found that CD with low CU showed deficits in recognizing fearful faces, while those with high CU showed deficits in recognizing sad faces (18, 22, 23). CU traits can also modulate the neural activity of emotion processing. Evidence showed that, activities in the amygdala for fear or distress stimuli decreased in CD with high CU, while they increased in CD with low CU (29–31). Another study revealed hyperactivity in prefrontal, posterior cingulate, and precuneal cortices among CD with high CU, compared with CD with low CU (24). As different types of CD may lead to different deficits in emotional processing, it would be a good test of validity for different theories. Nevertheless, more evidence should be collected to elucidate the effect of the type of CD on emotional processing more clearly.

Besides, more kinds of emotional cognition should be examined. Most studies till now concern the encoding processes of static emotional stimuli. First, other cognitive processes should also be examined. One important process might be emotional attention. It was revealed that adolescents with CD showed attentional avoidance and difficulty in attentional disengagement from facial expressions including angry, fearful, and happy faces (120). Another study found that adolescents with CD fixated less on the eyes of fearful and sad expressions (10). Other processes, such as memory and decision-making, require further investigations. Second, the deficits in dynamic emotional stimuli should be examined. It was found that, when viewing sad film clips, children with CD showed a larger increase in RSA than controls (121). However, when viewing an escalating conflict, male adolescents with aggressive CD showed less RSA reactivity than controls (94). There was also a study revealing that impaired emotional recognition in CD existed for both static and dynamic expressions (10). Taken together, a more general examination should be conducted on the emotional processing among CD to provide more evidence for the theories.

Finally, as the present review mainly focuses on emotional deficits and related theories among individuals with CD, other theories were not included. However, they should not be ignored or underestimated. For example, as impulsiveness is a typical characteristic of CD, the prefrontal dysfunction theory is critical in explaining the impulsiveness of CD. According to the prefrontal dysfunction theory, structural and functional deficits in the prefrontal lobe are associated with antisocial and aggressive behavior, because prefrontal dysfunction results in a lack of inhibitory control over antisocial and violent behaviors (32, 122). Interestingly, stimulation of the dorsolateral prefrontal lobe by tDCS (transcranial direct current stimulation) enhanced the activation in the prefrontal cortex and reduced aggressive intent (123). Therefore, prefrontal dysfunction theory is a successful theory concerning impulsiveness and aggression among CD. Further reviews may focus on other theories when discussing other deficits related to CD.
